# Evaluation of isolated abdominal visceral artery dissection with multi-scale spiral computed tomography: a retrospective case series

**DOI:** 10.1186/s13019-021-01428-8

**Published:** 2021-03-30

**Authors:** Qizhou He, Fei Yu, Yajun Fu, Bin Yang, Ran Huo, Rong Xian, Shulan Liu, Kali Liang, Guangcai Tang

**Affiliations:** 1 Department of Radiology, Affiliated Traditional Chinese Medicine Hospital of Southwest Medical University, Luzhou, 646000 Sichuan China; 2 Department of Radiology, Affiliated Hospital of Southwest Medical University, Luzhou, 64600 Sichuan China

**Keywords:** Dissection, Visceral artery, Computed tomography, Yun classification

## Abstract

**Background:**

To evaluate the role of multi-slice spiral computed tomography (MSCT) angiography in the diagnosis of spontaneous isolated visceral artery dissection (SIVAD).

**Methods:**

Twenty-seven patients with abdominal SIVAD were included in the study. The MSCT scans of the patients were subjected to various post-processing techniques to visualize the visceral artery wall. Clinical features including arterial dissection, thrombosis, dissection length, true/false lumen, and complications were recorded.

**Results:**

Type I, IIa, and IIb SIVADs were observed in 11, 6, and 10 patients, respectively. Superior mesenteric artery (SMA) dissection was the most common (*n* = 16), followed by abdominal aortic dissection (*n* = 6), splenic artery dissection (*n* = 2), renal artery dissection (*n* = 2), and splenic artery dissection (*n* = 1). One patient with SMA dissection suffered small intestine ischemia, 1 with splenic artery dissection had splenic infarction, and 1 patient with left renal artery dissection experienced renal infarction. The false lumen was bigger than the true lumen in 20 patients, with 9 patients having thrombus. The true lumen was bigger than the false lumen in 7 patients.

**Conclusions:**

MSCT angiography is a valuable technique in the diagnosis and treatment of patients with SIVAD. Patients with abdominal pain suspected due to SIVAD should be examined with MSCT angiography for early detection of SIVAD.

## Introduction

Spontaneous isolated visceral artery dissection (SIVAD), with and without aortic dissection, is rarely encountered in clinical practice. Most of the reported incidences of SIVAD are isolated case reports [1]. SIVAD includes spontaneous dissection of the mesenteric, celiac, splenic, and renal arteries [2, 3]. Its clinical features range from asymptomatic incidence to symptomatic cases including occlusion of the affected artery or dissecting aneurysm needing timely management to prevent acute catastrophic bowel ischemia or intestinal infarction [1, 4–6]. Majority of the previously reported cases of spontaneous isolated dissection are of the superior mesenteric artery (SMA), [1, 4, 6, 7] with a few being of the celiac artery [8–11]. Treatment strategies to manage SIVAD include conservative management (blood pressure control and bowel rest), use of anticoagulants, endovascular stenting, and surgical repair [5]. Previously, the incidence of SIVAD was underestimated because laboratory tests failed to detect it; however, with the advent and widespread use of multi-slice spiral computed tomography (MSCT), the number of reported incidents of SIVAD has increased, although it is still an uncommon (albeit important) diagnosis to make [7, 12, 13].

MSCT angiography is currently considered the preferred method for the diagnosis and follow-up of patients with isolated dissection of abdominal visceral artery [14–16]. This is because collimation width and computation time required have considerably reduced with the use of MSCT, and with thinner slices, it is possible to get increased coverage and higher resolution of image. A typical MSCT angiography image shows a double-lumen change or a low-density crescent-shaped filling defect in the artery, and a torn intimal flap between the false lumen and the true lumen. Given the limited clinical details available on SIVAD because of its rare occurrence, the therapeutic approach for treatment is still not standardized [17]. Using CT angiography, Yun et al. have classified lesions caused by SMA into the following types based on the cross-sectional and sagittal views: Type I, IIa, IIb, and III [6]. Correlating the clinical features of SIVAD with the MSCT imaging might help in understanding this disease along with the pathologies of various abdominal diseases including pancreatitis, abdominal pain, and abdominal distension. Therefore, this study retrospectively evaluated 27 Chinese patients with SIVAD who had undergone MSCT imaging. Clinical and imaging features of abdominal SIVAD were assessed to improve the understanding of this rare disease.

## Methods

### Study design

This was a single-center, retrospective study conducted at the Affiliated Traditional Chinese Medicine Hospital. Data were collected retrospectively for all consecutive patients with a diagnosis of SIVAD who were treated at our hospital between January 2014 and March 2017.

The included patients were either hospitalized because of clinical symptoms or were undergoing routine clinical examination from a single site. The study protocol was approved by the Institutional Review Board (IRB) of the Affiliated Traditional Chinese Medicine Hospital while conforming to the standards of the Declaration of Helsinki and its subsequent revisions. Only patients who had MSCT-confirmed SIVAD and those who signed informed consent were included in the study. Although informed consent was waived because of the retrospective nature of the study, as a general practice, a consent to use anonymized clinical information was collected at the time of hospitalization/clinical visit to all the included patients.

### MSCT imaging procedure

Patients were scanned using Siemens Definition Flash CT machine (Siemens healthcare) under the following settings: tube voltage: 120 KV; tube current: 300 mA; slice thickness: 5 mm; slice spacing: 5 mm; pitch: 0.6. All 27 patients underwent whole-abdomen CT enhancement with the non-ionic contrast agent Ultravist (370 mgI/L) (Bayer pharmaceuticals, Germany), 70 to 80 mL (1.2 mL/kg), which was injected using a double-barrel high-pressure syringe into the anterior elbow vein at a flow rate of 3.5 to 4.0 mL/s. This was followed by a 40-mL normal saline injection using a flushing catheter. The bolus was tracked with trigger scanning, for which the region of interest was set at the junction of the thoracic and abdominal aorta, and the threshold was 180 HU. The junction was scanned at the set threshold with a delay time of 25 to 28 s. The whole abdominal CT scan was completed while withholding 1 breath, and the original transverse image was obtained.

### Image post-processing

The original transverse image obtained with the enhanced MSCT scan was reconstructed with a slice thickness and slice spacing of 0.625 mm. The acquired data were further subjected to techniques including multi-planar recombination (MPR), surface reconstruction (CPR), volume rendering (VR), maximum density projection (MIP), and surface masking (SSD) to get the final processed MSCT angiographic images.

### MSCT image assessment and Yun classification

Two senior physicians were responsible for analyzing the MSCT images with abdominal vascular diagnosis. Observations from individual physicians were compared to ensure consistency. They evaluated the images for observing the (i) true and false double lumen of the abdominal visceral artery dissection (VAD), (ii) size of the true and false lumens, (iii) intimal flap, (iv) intimal rupture, (v) length of the dissection, (vi) thrombosis, (vii) presence or absence of visceral ischemia and infarction, and (viii) whether the branches were affected. In addition, the clinical symptoms observed were noted.

The physicians classified the vascular dissection based on Yun classification, [6] in which the isolated dissection of abdominal visceral artery is divided into 3 types: (i) Type I: true and false lumens showing entry and re-entry areas, (ii) Type II: true and false lumen but no re-entry of blood from the false lumen (IIa, false lumen without re-entry site; IIb, with thrombosis in false lumen leading to narrowing of the true lumen), and (iii) Type III: occlusion of the visceral artery.

## Results

### Demographics and patient characteristics

A total of 27 patients with abdominal SIVAD (age: 24–77 years; 24 men and 3 women) were included in this case series. Among the selected patients, only 3 were outpatients, whereas 24 were hospitalized. The causes for hospitalization included abdominal pain and/or abdominal distension (*n* = 18), lower back pain (*n* = 2), pain under the xiphoid process/precordial area (*n* = 1), and pain and discomfort in other body parts (*n* = 3; 1 each with axillary pain, esophageal cancer, and chest pain). The chief complaints of the outpatients included dysphagia (*n* = 1), bulging anal mass and prolapse (*n* = 1), and consciousness disorder (*n* = 1; Table 1).

**Table 1:** Clinical characteristics of the patients

**Location of dissection**	**Age**	**Artery Break**	**Length (mm)**	**Thrombosis**	**Large True Lumen**	**Large False Lumen**	**Yun Classification**	**Clinical Symptoms**	**Complications**
Left renal artery	49	yes	28	No	No	Yes	I	Left back pain for 8 days	No
Limited mesenteric artery	52	yes	15	No	Yes	No	IIa	stomach ache	Pancreatitis
Peritoneal cavity	63	yes	60	No	No	Yes	IIb	Abdominal pain for 20 days	No
Peritoneal cavity	46	yes	32	No	No	Yes	I	Abdominal pain for 20 h	Esophagus Cancer
Peritoneal cavity	77	yes	20	No	Yes	No	I	stomach ache	No
Peritoneal cavity	73	yes	14	No	Yes	No	IIa	Right chest and back pain for more than 1 month, aggravated for 1 day	No
Peritoneal cavity	61	yes	24	No	No	Yes	I	Consciousness disorder for 8 days, blackout for 11 h	No
Peritoneal cavity	67	yes	20	Yes	No	Yes	IIb	Physical examination found that the liver occupied 6 days	No
Peritoneal cavity and splenic artery dissection	68	yes	100	Yes	No	Yes	IIb	Mid-upper abdominal pain is 7 h	No
**Location of dissection**	**Age**	**Artery Break**	**Length (mm)**	**Thrombosis**	**Large True Lumen**	**Large False Lumen**	**Yun Classification**	**Clinical Symptoms**	**Complications**
Peritoneal cavity and splenic artery dissection	54	yes	25	No	Yes	No	IIa	Abdominal pain for 20 days	SMA branch and inferior mesenteric artery traffic
Proximal mesenteric artery	62	yes	35	No	Yes	No	I	Repeated anal mass prolapse for more than 1 year, aggravated with anal bulge in January	No
Right renal artery dissection	45	yes	15	Yes	No	Yes	IIb	Abdominal pain for more than 10 days	Iris tumor
SMA	47	yes	60	No	No	Yes	I	Precordial pain for 1 h	No
SMA	65	yes	60	No	No	Yes	I	Abdominal pain for 2 days	SMA and branch thrombus
SMA	47	yes	100	Yes	No	Yes	IIb	Repeated abdominal pain for 1 week	SMA thrombosis
SMA	53	yes	80	Yes	No	Yes	IIb	Abdominal pain for 12 h	No
SMA	64	yes	47	Yes	No	Yes	IIb	Dizziness with nausea for 15 h	No
**Location of dissection**	**Age**	**Artery Break**	**Length (mm)**	**Thrombosis**	**Large True Lumen**	**Large False Lumen**	**Yun Classification**	**Clinical Symptoms**	**Complications**
SMA	24	yes	30	No	Yes	No	IIa	stomach ache	Splenic artery thrombosis
SMA	44	yes	25	No	No	Yes	IIa	Mid-abdominal pain for 4 days	No
SMA	66	yes	95	Yes	No	Yes	IIb	Total abdominal pain for 6 h	No
SMA	77	yes	15	No	No	Yes	IIa	Repeated upper abdominal pain and discomfort for more than 10 years, recurrence increased by 10 days	Common hepatic artery originates from the false lumen
SMA	62	yes	70	No	No	Yes	I	Abdominal pain	No
SMA	51	no	130	No	No	Yes	I	Upper abdominal pain	No
SMA	65	yes	40	No	No	Yes	I	Abdominal pain	Postoperative dissection
SMA	55	yes	50	Yes	No	Yes	IIb	Abdominal pain	Pancreatitis
Splenic artery dissection	55	yes	25	No	Yes	No	I	Axillary pain for 1 year	No
Upper mesenteric artery/SMA	74	yes	60	Yes	No	Yes	IIb	Dysphagia - 3 days	No

*SMA* Superior Mesenteric Artery

### Angiographic characterization as per Yun classification

Using MSCT with the adjusted window width and window position technology, SIVAD can be visualized as a linear low-density shadow. Among the 27 patients evaluated, the physicians classified 11 patients as Type I, 6 as Type IIa, and 10 as Type IIb (Table 1). There were no Type III dissections observed in the studied population.

### Observed SIVAD types

**Fig. 1:**
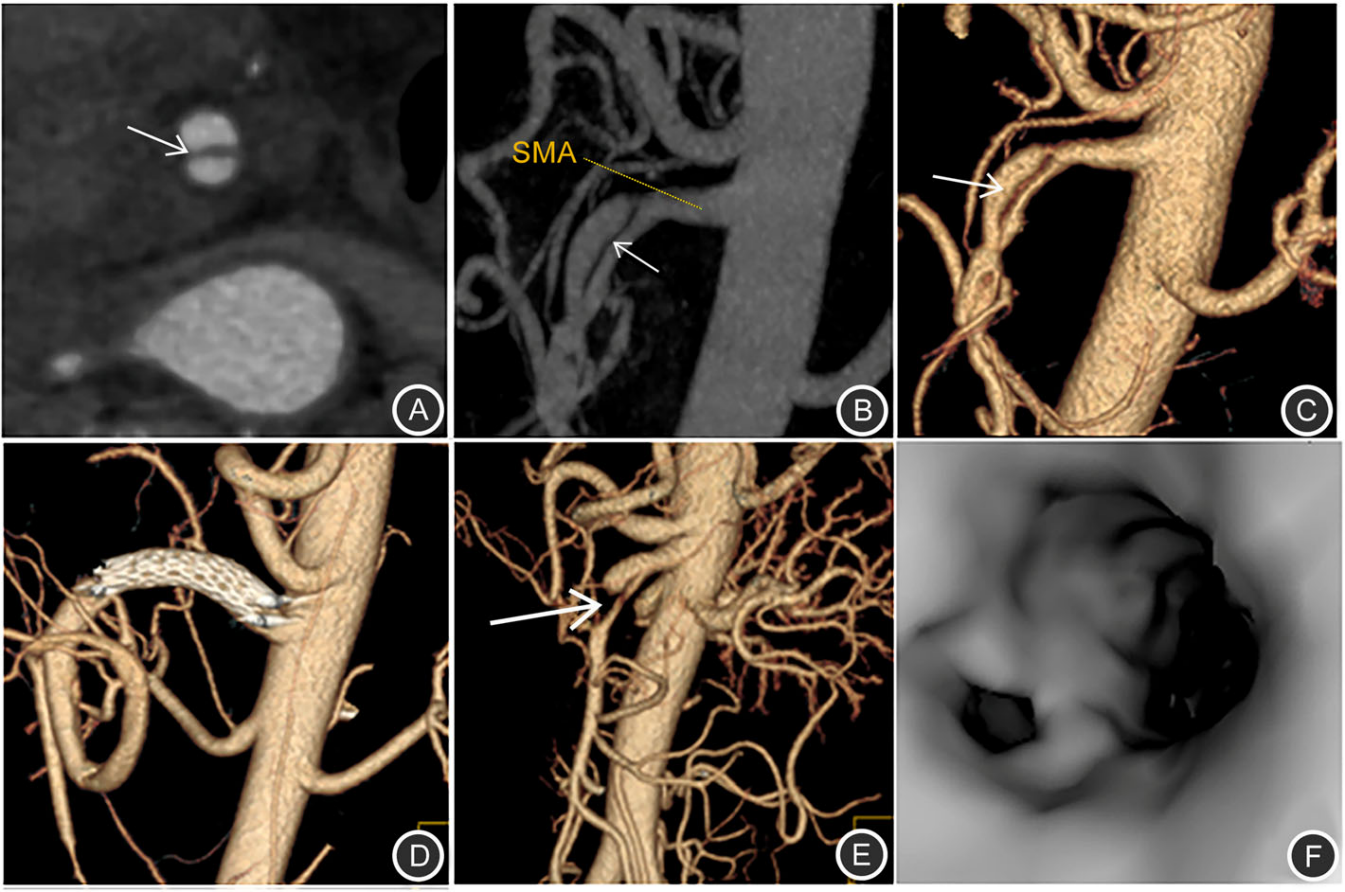
MSCT images of the superior mesenteric artery. **a** Axial image of the arterial phase shows the superior mesenteric artery inner intimal layer (arrow). The superior mesenteric artery is divided into 2 lm; **b**) MIP shows the superior mesenteric artery intimal flap and true and false double lumen and false lumen; **c**) VR shows the true and false double lumen; **d**) In the superior mesenteric artery intercalated with stent, VR clearly shows the position, length, and extent of the stent; **e**) VR shows the superior mesenteric artery prosthesis expansion and occlusion; **f**) CTVE clearly shows the superior mesenteric artery dissection

**Fig. 2:**
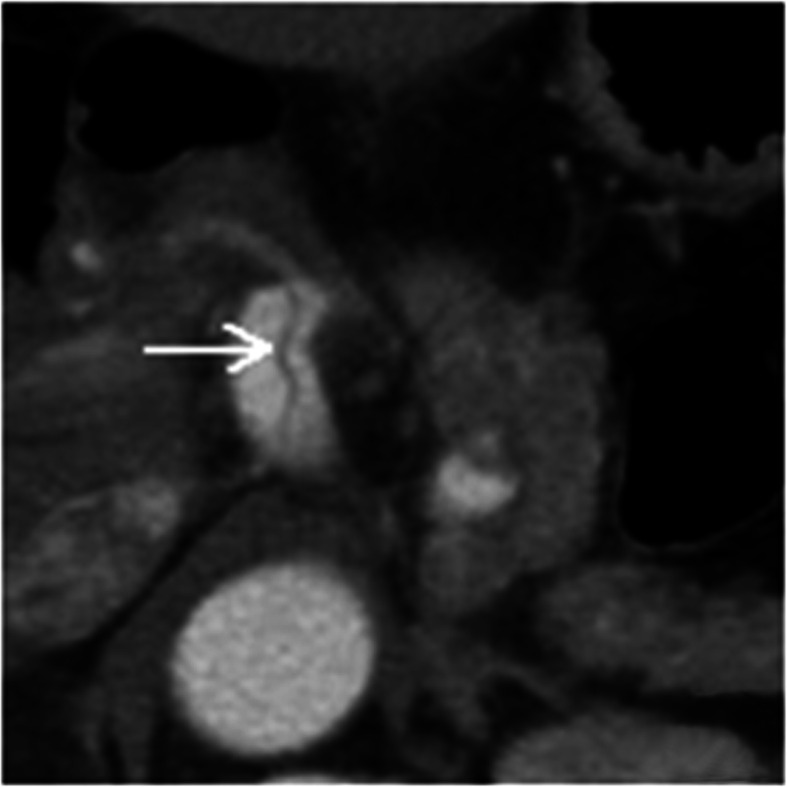
View through intra-abdominal low-density endocardial lens

**Fig. 3:**
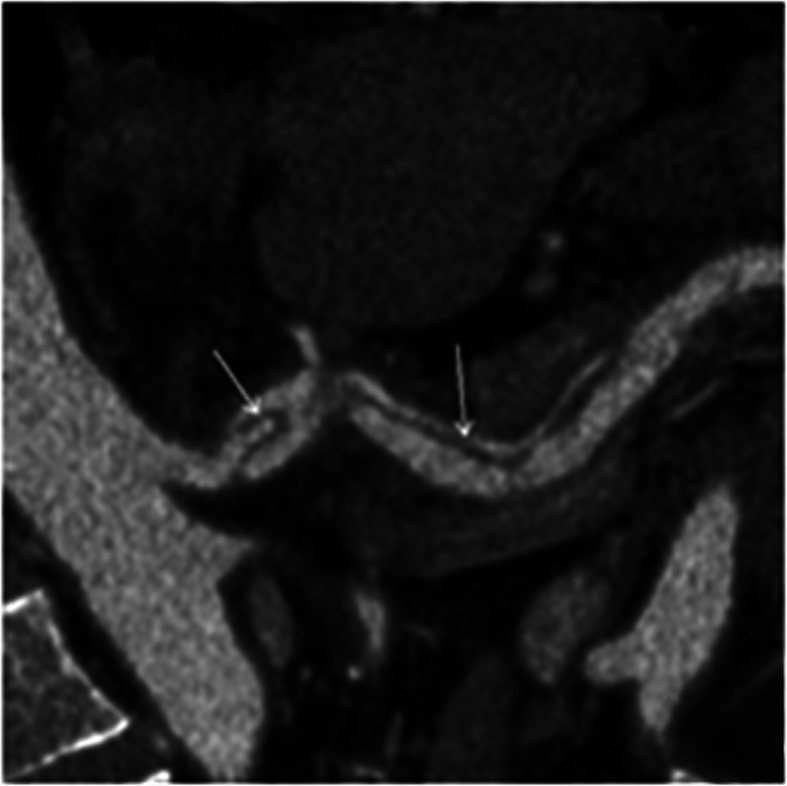
CPR image of the celiac trunk. White arrow: celiac trunk involved in the splenic artery

**Fig. 4:**
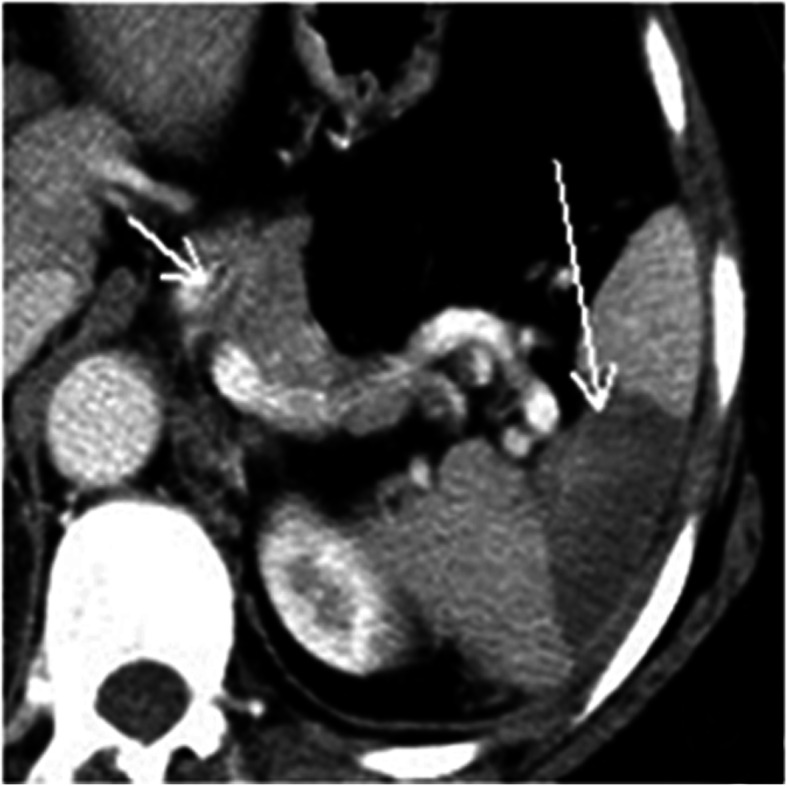
Splenic artery dissection with spleen infarction. Arrows indicate splenic artery dissection and infarction

**Fig. 5:**
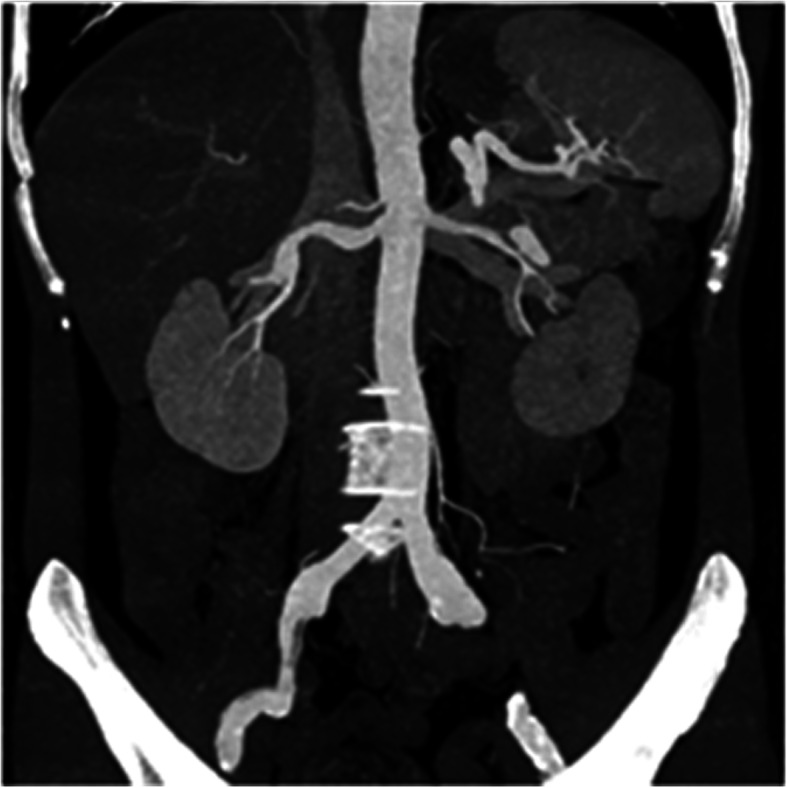
VR shows renal artery dissection

A total of 16 patients with SMA dissections were encountered (Figs. 1 and 2). The other types of dissections observed were isolated abdominal aortic dissection (*n* = 6), splenic artery dissection (*n* = 2, Fig. 3) involving abdominal aorta due to splenic intramural thrombus, splenic artery dissection (*n* = 1; Table 1, Fig. 4), and renal artery dissection (*n* = 2; Table 1, Fig. 5). After processing the MSCT images with MPR, VR, MIP, and CPR, the visceral artery wall was clearly viewed in both the true and false double-chambered and exposed intimal layer. A total of 20 patients had intimal rupture, with a wall length in the range of 14 to 130 mm (Table 1). About 74.1% patients were observed to have a larger false lumen compared with the true lumen, whereas 25.9% patients had a larger true lumen compared with the false lumen. An additional 4 patients had true lumen stenosis, whereas thrombus in the false lumen was observed in 9 patients. Blood supply was observed in 14 patients with arterial dissection, and blood in the false lumen was observed in 3 patients.

### Other associated clinical conditions and suspected complications of SIVAD

The other clinical conditions associated with abdominal VAD observed in the study included cancer of the esophagus and iris (*n* = 1, each), which were chance findings. Further, 2 patients with type IIA and IIB SIVAD had pancreatitis (Table 1). One patient with SMA dissection suffered small intestine ischemia, 1 with splenic artery dissection had splenic infarction, and 1 patient with left renal artery dissection experienced renal infarction.

## Discussion

Unlike common reports of carotid dissection, [18–22] SIVAD is rarely reported [8–11, 23] and is associated with multiple serious consequences such as abdominal splanchnic artery aneurysm, dilation and rupture leading to intra-abdominal hemorrhage, vital organ ischemia, and SMA/renal/splenic artery embolization [24]. Owing to the scarcity of the data available, the underlying cause of the disease and pathogenesis remain unclear. MSCT, with the high-quality images produced in a time-efficient manner, could provide better insight into the clinical conditions associated with SIVAD [25]. This study evaluated the association of features on high-quality MSCT scan images with the clinical symptoms of SIVAD. For most of the patients included in the study, SIVAD was diagnosed based on characteristic MSCT findings such as intramural hematoma, thrombosis of false lumen, and/or intimal flap [13, 26]. The results obtained showed that MSCT scan, along with various post-processing techniques (MPR, CPR, VR, MIP, and SSD), can provide accurate diagnosis and aid in understanding the implications of arterial dissection.

Previously, Sakamoto et al. had classified the spontaneous dissection of the SMA for the first time into 4 types based on the imaging features [27]. This classification was further supplemented and modified by Yun et al. [6] In this study, most of the patients had SIVAD Type I (*n* = 11, 40.74%), followed by Type IIb (*n* = 10, 37.04%) and Type IIa (*n* = 6, 22.22%) according to Yun classification. These observations are in line with the findings of Yun et al. who reported Type II as the most common type of spontaneous artery dissection (Type I: 41%; Type II: 50%: Type III: 9%) [6].

The first dissection of the SMA wall is usually 1.5 to 3.0 cm from the beginning of the artery, that is, the transition point between the fixed and free segments of the SMA [6, 28]. This could be because at this site, the curvature of the blood vessel wall is larger, and hence, the higher blood shear stress causes the membrane tear [29, 30]. Yun et al. reported that the dissection length is positively associated with the severity of the clinical symptoms (*P* = 0.03) [6]. However, the study by Yun et al. only included patients with SMA dissection, whereas the present study included patients with spontaneous isolated dissection of the splenic artery, celiac artery, and left renal artery along with patients with SMA dissection.

In patients with Type I spontaneous arterial dissection, the hemodynamics is almost unaffected owing to the presence of relatively stable and unobstructed false-lumen, and hence, this type can be treated with best efficacy. There were 8 patients with Type I dissection among the included patients with no complications. In patients with Type II arterial dissection (both IIa and IIb), for whom true lumen is unobstructed and false lumen is not smooth, conservative treatment may effectively relieve symptoms at an early stage [13]. However, as blood flow in the false lumen is poor, some of the false lumen blood flow can fail to flow out, resulting in higher tension inside the lumen that can further increase the risk of dissection. In this study, 6 patients had Type IIa dissection, of which 1 patient had spleen infarction, 1 patient suffered renal infarction, and the remaining 3 patients were free from any complication. A total of 10 patients with Type IIb dissections were accompanied by thrombosis of the false lumen. Partial or complete thrombosis of the false lumen has been previously reported as a factor predicting mortality in patients with type B aortic dissection and SIVAD [31, 32].

Furthermore, 2 patients with type IIa and IIb, isolated SMA dissection in this study had acute pancreatitis. It has been reported that the pancreatic enzymes released during pancreatitis can erode adjacent arterial wall and thus, acute pancreatitis might have positively influenced arterial dissection [33, 34]. In addition, 2 patients had renal artery dissection with kidney stones, which is consistent with the findings of Luan et al., who reported the presence of kidney stones in 5 of the 622 patients evaluated [7]. Moreover, 18 patients had only abdominal pain of varying duration and intensity, whereas the remaining 9 patients had atypical clinical manifestations. The pain in the upper abdominal area is caused by the tearing of the artery wall’s intima and the increased pressure of the false lumen [14]. Moreover, the increase in fat density around the arteries and the inflammatory response induced by the dissection can stimulate abdominal pain via the visceral nerve plexus, and the degree of pain is positively correlated with the length of the dissected blood vessel [6, 35]. For the 3 patients with reported organ infarction or ischemia, we speculate that increased tension and oppression of the true lumen might have obstructed the local blood flow in the organ, leading to organ infarction.

There are various factors considered to be associated with the incidence of spontaneous dissection of the visceral artery including hypertension, age, gender, connective tissue diseases, vasculitis, atherosclerosis, arterial cystic necrosis, trauma, iatrogenic injury, pregnancy, smoking, autoimmune diseases, and tumors [7, 12, 13, 23, 36, 37]. Our findings that 8 of the included patients were hypertensive, older, and partially atherosclerotic are consistent with these previous reports. We speculate that abnormal hemodynamic changes caused by hypertension could have caused changes in arterial function and structure, which might have eventually resulted in arterial dissection [30]. In addition, intracavitary thrombosis and higher fat deposition around the arteries could predispose the artery to spontaneous dissection [8, 13, 38]

For the diagnosis and follow-up of abdominal SIVAD, digital subtraction arteriography (DSA), magnetic resonance angiography (MRA), and MSCT are currently in use [39, 40]. However, DSA has certain limitations. It can only visualize the lumen filled by the contrast agent, and false lumen containing blood clots may not be appreciated [41]. Further, DSA is an invasive, high-risk, and expensive technique with relatively low specificity and sensitivity. On the other hand, MRA is helpful in diagnosing the arterial dissection but requires longer examination time, and the clarity of image is negatively affected by the small lumen of the visceral arteries and peristaltic movements in the abdomen of patients with clinical abdominal pain. Hence, MSCT angiography has become the preferred method as it accurately displays the location, range, and collateral distributions of the arterial dissection, as well as indicates secondary changes including necrosis and perforation in abdominal organs. Details procured with MSCT angiography can help diagnose the disease, monitor postoperative treatment effects, and provide a better understanding of the pathological changes that occur during SIVAD [14, 24, 42]. Because of the small diameter of the abdominal visceral artery, the CT image of the abdominal cross-section obtained with the window width technique is often not clear. The sleek dual-source CT overcomes the limitation of window width with its large scanning range and shorter scanning time, and the image obtained can be reconstructed in various ways. Therefore, the authors recommend that for patients experiencing sudden and significant abdominal pain with no specific disease related symptoms, enhanced MSCT imaging should be performed to evaluate the abdominal visceral arteries.

Our study had a few limitations. First, this was a retrospective study, which might have led to unintentional bias during data extraction and reporting. Second, the patients were selected from a single center and in a small number; therefore, the results might be difficult to generalize in a larger number of patients. Evaluation of a larger population could have provided a more robust insight in to the disease. Third, the patients were not followed up to assess the effect of treatments on outcomes. Finally, the observations made with MSCT angiography were not compared with those obtained with other techniques such as DSA.

## Conclusion

To conclude, abdominal SIVAD is a commonly misdiagnosed condition that can be accurately and effectively diagnosed using MSCT angiography in combination with post-treatment methods, which will allow to understand the overall process of arterial dissection. In addition, MSCT images can guide the treatment and prognosis of abdominal SIVAD.

## Data Availability

All data associated with the study have been included in this manuscript.

## Abbreviations

MSCT: Multi-slice spiral computed tomography
SIVAD: Spontaneous isolated visceral artery dissection
SMA: Superior mesenteric artery
MPR: Multi-planar recombination
CPR: Surface reconstruction
VR: Volume rendering
MIP: Maximum density projection
SSD: Surface masking
DSA: Digital subtraction arteriography
MRA: Magnetic resonance angiography
